# The chromatin remodelling factor Chd7 protects auditory neurons and sensory hair cells from stress-induced degeneration

**DOI:** 10.1038/s42003-021-02788-6

**Published:** 2021-11-03

**Authors:** Mohi Ahmed, Ruth Moon, Ravindra Singh Prajapati, Elysia James, M. Albert Basson, Andrea Streit

**Affiliations:** 1 Centre for Craniofacial and Regenerative Biology, Floor 27 Tower Wing, Guy’s Hospital, King’s College London, London, SE1 9RT UK; 2 Wolfson Centre for Age-Related Diseases, Institute of Psychiatry, Psychology and Neuroscience, King’s College London, London, SE1 1UL UK; 3 MRC Centre for Neurodevelopmental Disorders, King’s College London, London, SE1 1UL UK; 4 Present Address: Leukaemia and Stem Cell Biology Group, School of Cancer and Pharmaceutical Sciences, King’s College London, London, SE5 9NU UK

**Keywords:** Neuroscience, Disease model

## Abstract

Neurons and sensory cells are particularly vulnerable to oxidative stress due to their high oxygen demand during stimulus perception and transmission. The mechanisms that protect them from stress-induced death and degeneration remain elusive. Here we show that embryonic deletion of the chromodomain helicase DNA-binding protein 7 (CHD7) in auditory neurons or hair cells leads to sensorineural hearing loss due to postnatal degeneration of both cell types. Mechanistically, we demonstrate that *CHD7* controls the expression of major stress pathway components. In its absence, hair cells are hypersensitive, dying rapidly after brief exposure to stress inducers, suggesting that sound at the onset of hearing triggers their degeneration. In humans, *CHD7* haploinsufficiency causes CHARGE syndrome, a disorder affecting multiple organs including the ear. Our findings suggest that *CHD7* mutations cause developmentally silent phenotypes that predispose cells to postnatal degeneration due to a failure of protective mechanisms.

## Abstract (web summary)

To improve our understanding of the mechanisms that protect hair cells in the ear from stress-induced death, Ahmed et al delete the chromodomain helicase DNA-binding protein 7 (CHD7) in auditory neurons and hair cells in mice. They observe sensorineural hearing loss and demonstrate that CHD7 controls the expression of stress pathway components, which could help to explain how CHD7 haploinsufficiency causes changes in the ear associated with CHARGE syndrome.

## Introduction

Sensorineural hearing loss (SNHL) is a common feature of CHARGE syndrome and affects 50–70% of individuals^1,2^. Mice with heterozygous *Chd7* mutations are an excellent model for CHARGE syndrome and, like humans, they exhibit SNHL^3–6^. *Chd7* plays an important role during neurogenesis both in the brain and the inner ear^5,7–12^. At embryonic day (E) 9.5-E10.5, neuronal progenitors are reduced in the inner ear of *Chd7*^*+/−*^ mutants, and Chd7 is necessary for their proliferation^5^. However, by E11.5, the number of neuronal progenitors is restored and inner ear neurons, as well as the hair cells they innervate, appear normal after birth^5,6^. Therefore, the cellular function of *Chd7* and the mechanisms underlying SNHL have yet to be elucidated.

In the cochlea, inner hair cells are responsible for sound perception, while outer hair cells modulate the sound amplitude^13^. They are innervated by type I and type II spiral ganglion neurons, respectively, which project to the auditory nuclei in the brain stem^14^. As the hair cells (and spiral ganglion neurons) lack the capacity to regenerate, oxidative stress caused by loud noise, ageing, or ototoxicity leads to cell death and permanent hearing loss^15–19^.

## Results

**Fig. 1:**
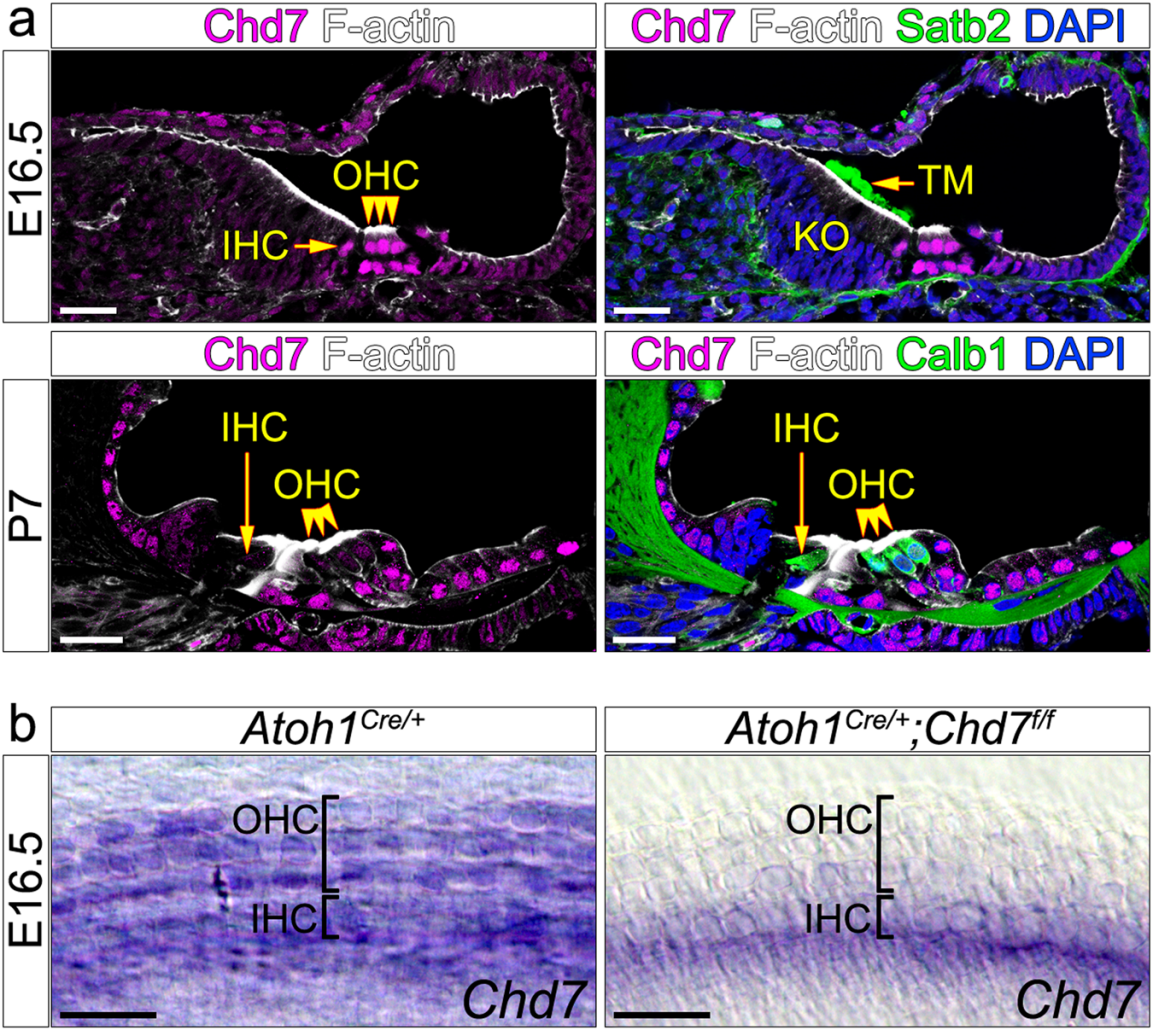
Chd7 expression in wildtype and *Atoh1*^*Cre/+*^*;Chd7*^*f/f*^ mutant organ of Corti. **a** Immunohistochemistry in wildtype cochlea showing Chd7 expression in hair cells at E16.5 and P7. Expression is weaker at P7 compared to E16.5. Hair cells are stained with F-actin and Calbindin 1. Tectorial membrane is stained with Satb2. **b** In-situ hybridisation showing loss of *Chd7* expression in *Atoh1*^*Cre/+*^*;Chd7*^*f/f*^ mutant hair cells within the middle region of the cochlea at E16.5. *Atoh1*^*Cre/+*^ or *Chd7*^*floxed*^ siblings were used as controls (indicated at the top of the panel). IHC inner hair cells; OHC outer hair cells; TM tectorial membrane. Scale bars 25 µm.

In mice, hair cell specification mediated by the transcription factor Atoh1 occurs between E12.5 and E16.5^20,21^. However, their development continues postnatally^20^ and they reach maturity just before the onset of hearing between postnatal day 10 (P10) and P14^22^. Chd7 is expressed in hair cells and neurons throughout embryonic and postnatal stages (Fig. 1a, Fig. S1)^3,5,6^. To investigate its function in hair cells, we deleted *Chd7* using the hair cell-specific *Atoh1*^*Cre*^ driver (*Atoh1*^*Cre/+*^*;Chd7*^*flox*^). We compared Cre-positive and Cre-negative control with *Chd7* heterozygous and homomozygous littermates. In *Atoh1*^*Cre/+*^*;Chd7*^*flox*^ mice, *Chd7* deletion was initiated in hair cells from E12.5 onwards and we confirmed loss of *Chd7* expression in the basal and middle turns of the cochlea at E16.5 (Fig. 1b). Surprisingly, hair cells appeared normal at P8 (*n* = 10/10; Fig. S2a, b) suggesting that Chd7 function may not be required for their development.

**Fig. 2:**
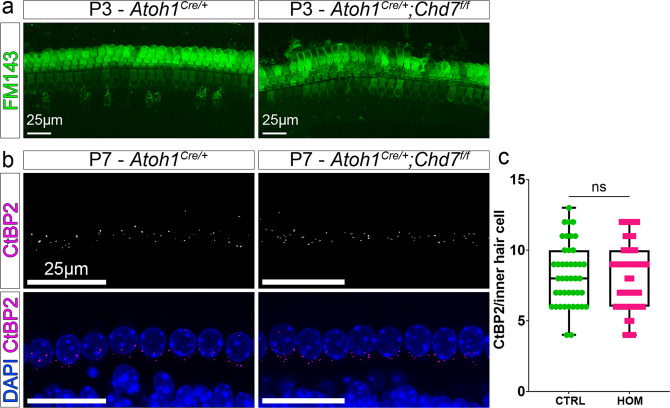
Hair cell development is not affected by Chd7 deletion. **a** Cochlear explants of control and *Chd7* homozygous mutants were treated with FM1-43 for 30 s to assess mechanotransduction. **b** CtBP2 immunohistochemistry of control and *Chd7* homozygous mutants at P7 shows that ribbon synapses at the inner hair cells are unaffected in mutants. **c** Quantification of CtBP2 puncta (ribbon synapses) per inner hair cell. CTRL = control; HOM = homozygote.

We, therefore, wished to confirm hair cell integrity and functionality in the early postnatal cochlea. A key feature of hairs cells is their ability to transform mechanical force into electrical energy that in turn elicits action potentials in spiral ganglion neurons. This requires functional mechanotransduction channels located in the hair cell stereocilia^23,24^. We visualised uptake of the styryl membrane dye FM1-43 through these channels to assess their activity. Cochleae isolated from P3 mice were exposed to FM1-43 for 30 s and imaged using confocal microscopy. Hair cells from both *Atoh1*^*Cre/+*^ (or *Chd7*^*f/f*^) control and *Atoh1*^*Cre/+*^*;Chd7*^*f/f*^ homozygous mutant mice showed robust FM1-43 uptake, indicating that mechanotransduction in mutant hair cells was relatively normal (Fig. 2a). To investigate whether mutant inner hair cells established connectivity with spiral ganglia neurons, we assessed the presence and density of ribbon synapses; there was no difference between controls and mutants at P7 (Fig. 2b, c). Together, these results suggest that although *Chd7* is deleted at embryonic stages as hair cells are specified, hair cells develop with normal morphology until the first postnatal week with intact mechanotransduction channels and neuronal connections.

**Fig. 3:**
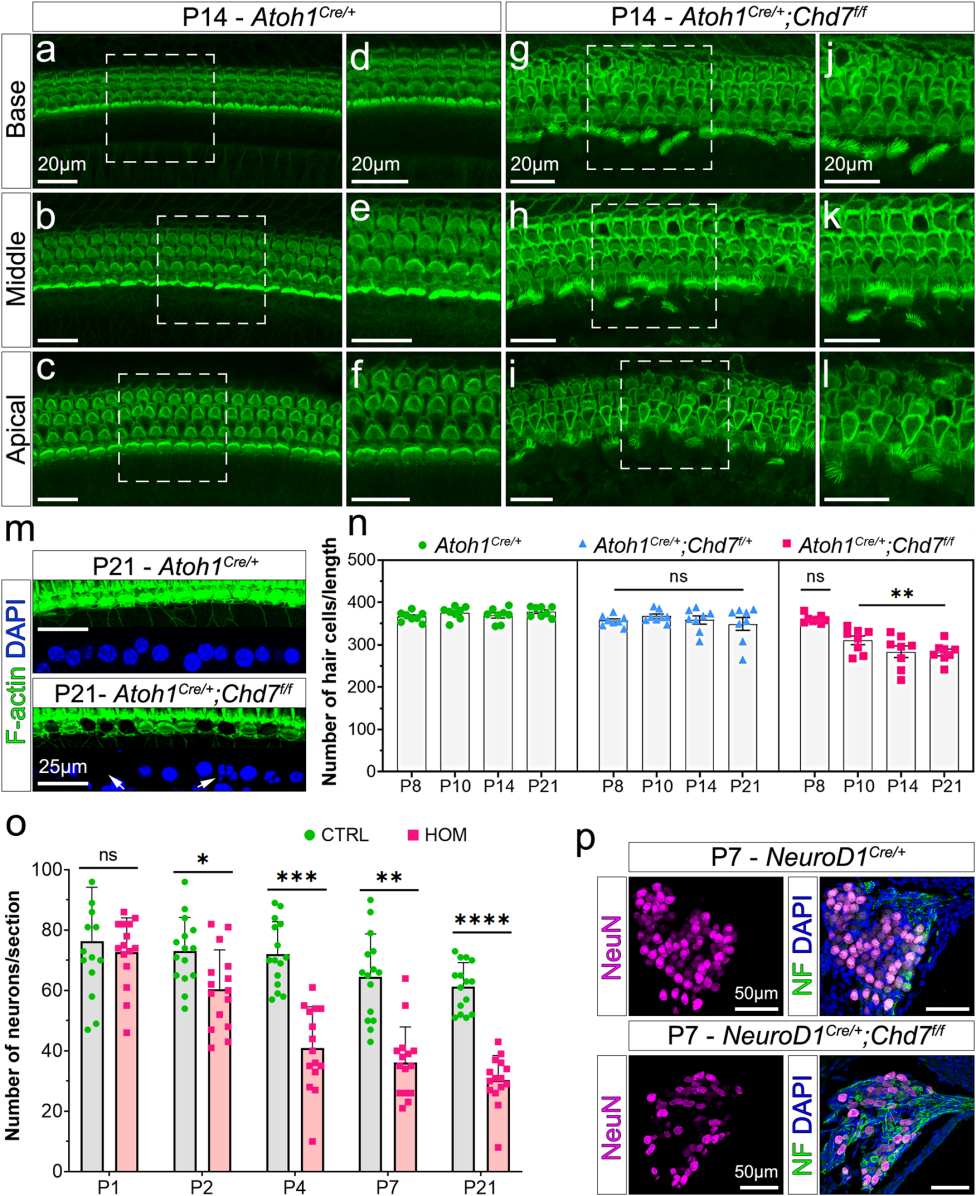
Postnatal degeneration of hair cells and neurons in *Chd7* mutants. **a**–**l** F-actin-stained hair cells in the cochlea of control (**a**–**f**) and *Chd7* homozygous mutants (**g**–**l**) at P14. Dashed boxes in **a**–**c** and **h**, **i** indicate the zoomed regions shown in (**d**–**f**) and (**j**–**l**). Scale bars = 20 µm. **m** Inner hair cells showing pyknotic, fragmented (arrow) and missing (arrow) nuclei in *Chd7* mutants at P21. Scale bars = 25 µm. **n** Average number of hair cells per 200 µm regions in base, middle and apical turns of each cochlea per animal (*n* = 8 per genotype; each animal is represented by one circle, triangle or square). Separate inner and outer hair cell quantification is provided in Fig. S2. Statistical significance was obtained by performing a nested one-way ANOVA and Dunnett’s multiple comparison test. **P-value = 0.005. **o** Average number of neurons in the spiral ganglion per section at different postnatal stages in control (CTRL) and *Chd7* homozygous mutants (HOM). Student’s *t* test, *P*-values: *= < 0.05, **< 0.005, ***= < 0.0005, ****= < 0.00005). **p** Spiral ganglia neurons stained with NeuN and neurofilament (NF) at P7 in control and mutant animals. Scale bars = 50 µm.

Thereafter we observed rapid degeneration of inner hair cells at P10 (*n* = 6/8) and P14 (*n* = 15/16), while outer hair cells degenerated more slowly (Fig. 3a–l, n, Fig. S2a, b). By P21, most inner hair cell nuclei were missing, pyknotic or fragmented, indicating progressive degeneration and cell death (*n* = 8/8; Fig. 3m, n, Fig. S2b). *Chd7* heterozygous mutants showed equally severe phenotypes (Fig. S2c) but at a reduced frequency (*n* = 5/24). To establish when *Chd7* function is critical during hair cell formation, we used inducible *Atoh1*^*CreERT2*^ to temporally delete *Chd7*. Tamoxifen was administered at E16, with loss of Chd7 protein, particularly in inner hair cells, evident at E18.5 (Fig. S3a, b). Unlike early embryonic *Chd7* deletion as soon as hair cells were specified, loss of *Chd7* after E16.5 did not cause postnatal hair cell degeneration (*n* = 6/6; Fig. S3c). These observations suggest that *Chd7* loss during early hair cell development predisposes cells to postnatal degeneration which leads to SNHL.

Postmitotic neural progenitors arise in the otic vesicle from ~E9 onwards under the control of NeuroD1 and differentiate into spiral ganglion neurons by E14.5^25–28^. However, the peripheral auditory circuit is only established in the first 10 days after birth (P0-P10), prior to the onset of hearing^14,29,30^. To assess *Chd7* function in spiral ganglion neurons, we analysed *NeuroD1*^*Cre/+*^*;Chd7*^*floxed*^ mutants. In homozygous mutants, ganglion size and neuronal numbers were indistinguishable from controls at P1 (*n* = 3/3; Fig. 3o, Fig. S4a), but neurons degenerated rapidly to 50% by P7 (*n* = 3/3; Fig. 3o, p). This phenotype was also observed in *Chd7* heterozygous mutants, although neurodegeneration occurred gradually with 50% loss by P21 (*n* = 3/3; Fig. S4b). Thus, *Chd7* controls the survival of a subset of spiral ganglion neurons. Like in hair cells, *Chd7* deletion at embryonic stages does not appear to affect neuronal development but leads to delayed neurodegeneration postnatally.

**Fig. 4:**
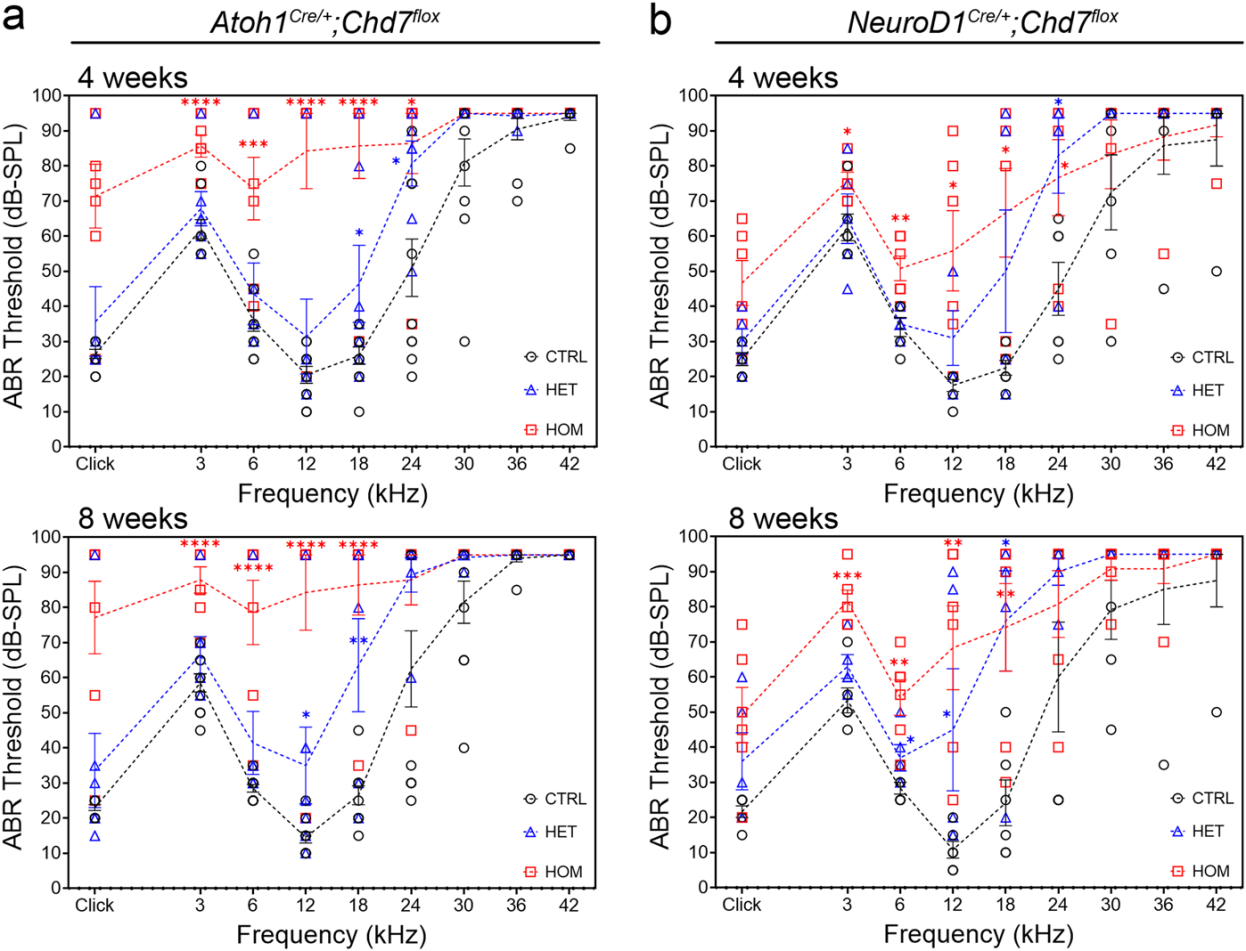
Postnatal degeneration of hair cells and neurons in *Chd7* mutants. **a** Auditory brainstem response (ABR) tests of *Atoh1*^*Cre/+*^*;Chd7*^*flox*^ mutants and controls at 4 weeks and 8 weeks reveals profound hearing loss in homozygous and mild-moderate hearing loss in heterozygous *Chd7* mutants across all frequencies. **b** ABR tests of *NeuroD1*^*Cre/+*^*;Chd7*^*flox*^ mutants and controls at 4 weeks and 8 weeks reveals mild-moderate hearing loss in homozygous mutants. Frequencies where significant threshold elevations were observed are indicated by asterisks (*P*-values: *< 0.05, **< 0.005, ***< 0.0005, ****< 0.000005). See Figs. S5 and S6 for ABR profiles of each mouse. Error bars represent the standard error of mean (see Figs. S5 and S6). CTRL = control; HET = heterozygote; HOM = homozygote.

To determine whether *Chd7* deletion results in hearing loss, we measured the auditory brainstem response (ABR) of 4- and 8-week-old mutant and control animals. Most *Atoh1*^*Cre/+*^*;Chd7*^*flox*^ homozygous mutants exhibited severe-profound hearing loss across all frequencies (*n* = 6/7; Fig. 4a, Fig. S5), while only 1/7 heterozygous mutants showed a similar ABR profile (Fig. S5). In contrast, *NeuroD1*^*Cre/+*^*;Chd7*^*flox*^ mutants exhibited moderate hearing loss (*n* = 6; Fig. 4b, Fig. S6), presumably due to surviving neurons. Nonetheless, the ABR tests confirm that SNHL correlates with hair cell or neuronal degeneration.

**Fig. 5:**
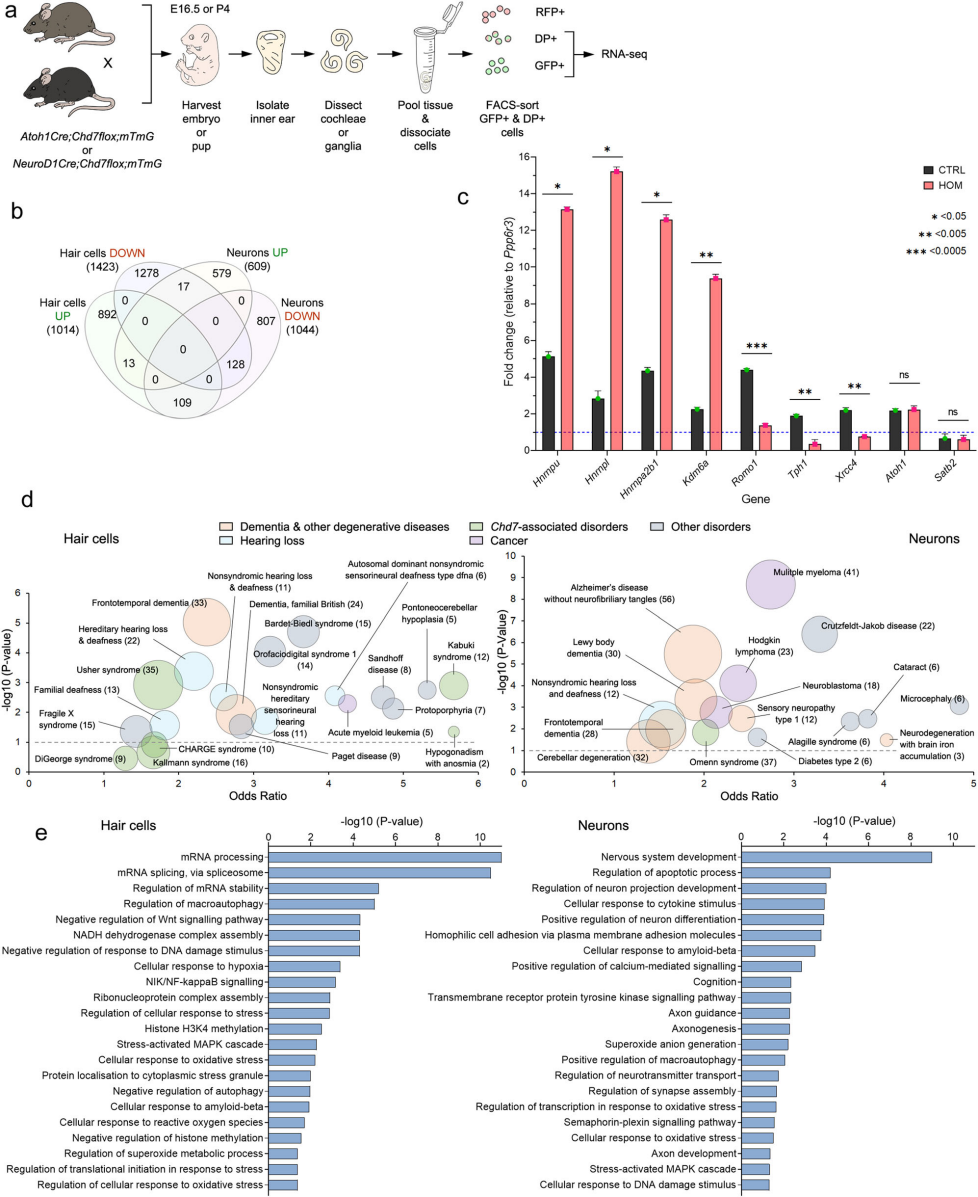
Transcriptome analysis of control and *Chd7* mutant hair cells and neurons reveals misregulation of cellular stress pathways. **a** Schematic showing the experimental approach used for RNA sequencing. DP = double positive. *n* = 6 cochleae or ganglia were pooled for RNA-seq in three independent experiments per genotype. **b** Comparison of the number of differentially expressed genes between hair cells and neurons in homozygotes. **c** qPCR expression analysis of genes in controls and homozygous FAC-sorted hair cells at E16.5. Error bars represent the standard error. *P*-values: *= < 0.05, **< 0.005, ***< 0.0005. ns = not significant. **d** Plot of Odds ratio by −log10 of the *P*-value for human diseases identified by disease ontology. Odds ratio is used to determine the relative odds of the occurrence and magnitude of disease, given exposure to the risk factor (e.g., genes). Complete disease ontology is provided in Supplementary Data 5 and 6. **e** Gene ontology for differentially regulated genes. Complete gene ontology is provided in Supplementary Data 7 and 8.

Chd7 controls transcription through regulation of chromatin architecture^9,11^, but how it exerts its function in auditory hair cells and neurons is poorly understood. We, therefore, examined the earliest transcriptional changes resulting from *Chd7* deletion by comparing gene expression of FAC-sorted hair cells or spiral ganglia neurons from mutant and control animals (Fig. 5a, Supplementary Data 1, 4). Using the non-inducible (*Atoh1*^*Cre/+*^) and inducible (*Atoh1*^*CreERT2/+*^) mediated deletion of *Chd7* enabled us to pinpoint the earliest developmental window in which Chd7 function is required (i.e., between E12.5 and E16.5). By E16.5, most hair cells have formed^20,21^; by this time point *Chd7* expression was lost in mutant cochleae. Therefore, we analysed changes in hair cell gene expression at E16.5. We were unable to determine the precise time point for Chd7 function in neurons due to the lack of an inducible *NeuroD1*^*CreERT2/+*^ transgenic line. We, therefore, chose P4 for transcriptional profiling to capture the time point when spiral ganglion neurons actively undergo cell death in mutants (Fig. 3o) and when they normally establish synaptic connections with hair cells^14,29,30^.

Differential gene expression analysis revealed significant changes (FDR ≤ 0.05, fold change >2) in 2437 transcripts in hair cells and 1653 transcripts in neurons (Fig. 5b, Fig. S7a–d, Supplementary Data 1–4). We selected genes with high fold-change in expression (see Supplementary Data 2) as well as genes unaffected by *Chd7* deletion (e.g., *Atoh1*) and transcripts that are normally not expressed (e.g., *Satb2*) for validation by qRT-PCR (Fig. 5c) and protein by immunohistochemistry (Fig. S8). These results confirmed our RNAseq data. Analysis of Disease Ontology terms for all differentially expressed genes showed enrichment of *Chd7*-associated syndromes as well as hearing loss. Surprisingly, there was also a strong association with neurodegenerative diseases including dementia (Fig. 5d, Supplementary Data 2, 4, 5, 6) pointing towards a common mechanism underlying these conditions.

**Fig. 6:**
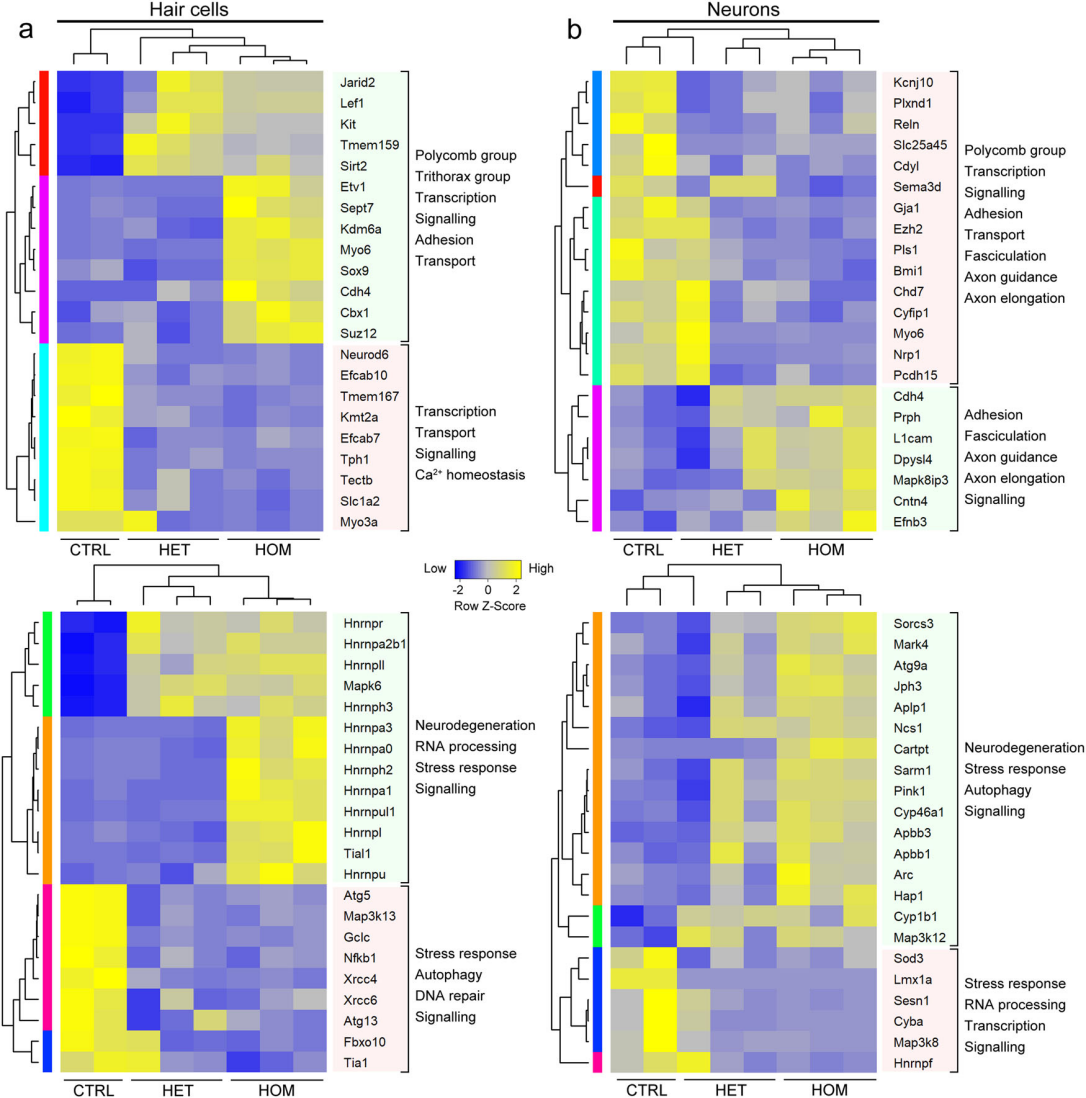
Chd7 regulates RNA-splicing and stress pathway genes. **a** Heatmap of representative functional categories of differentially expressed genes in hair cells. **b** Heatmap of representative functional categories of differentially expressed genes in neurons. Highlighted blocks of genes: green = upregulated; red = downregulated.

**Fig. 7:**
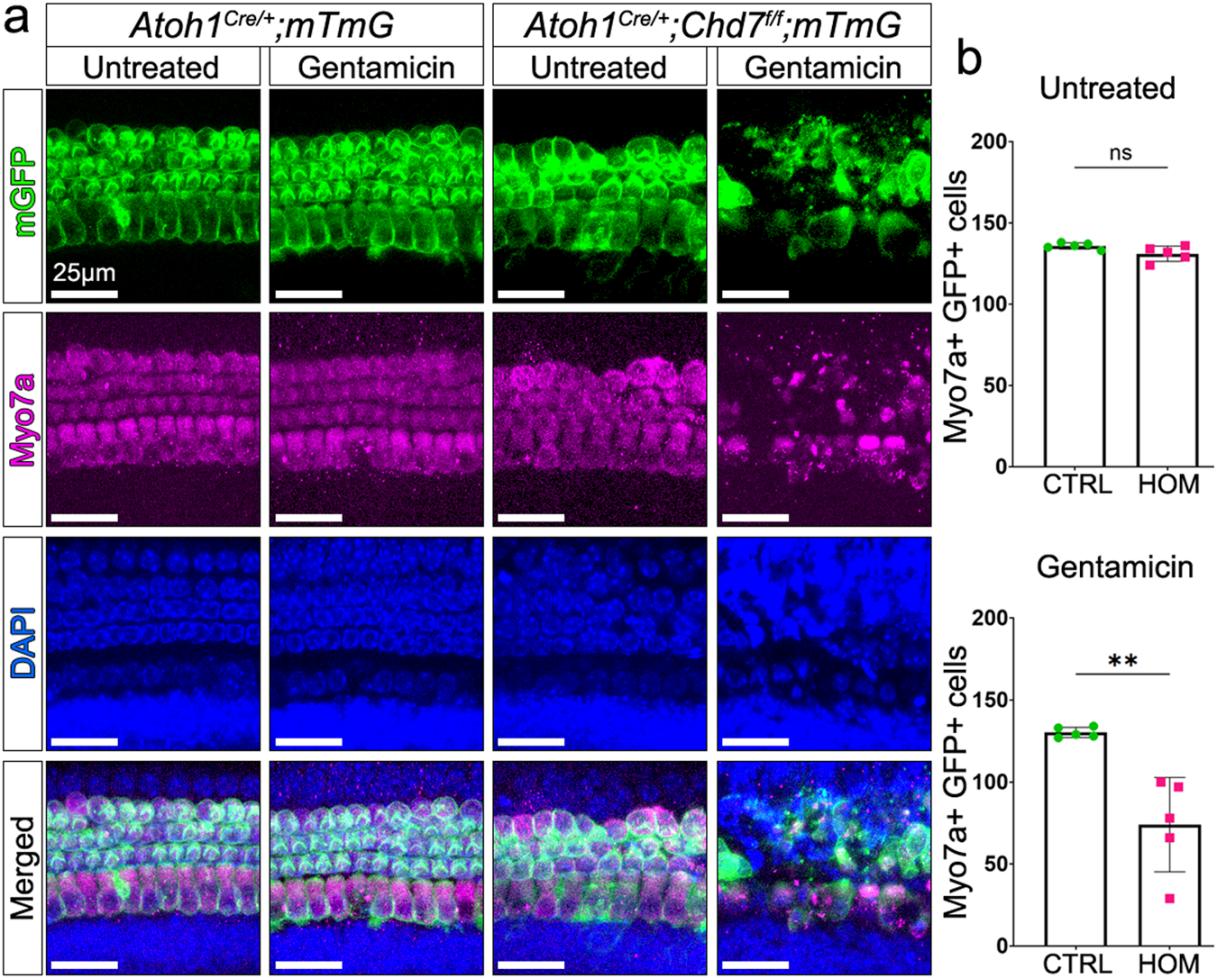
*Chd7* mutant hair cells are hypersensitive to stress. **a** Cochlear explants of control and *Chd7* homozygous mutants were treated with gentamicin to induce oxidative stress. Rapid hair cell death is observed in mutants within 5 h whereas control hair cells and untreated mutant hair cells survive. Green = Cre recombined cells expressing membrane GFP, magenta = all hair cells stained with Myo7a, blue = DAPI stained nuclei. **b** Quantification of Myo7a+ and GFP+ hair cells per 200 µm region in untreated and treated explants in both controls (CTRL) and mutants (HOM). Two-tailed unpaired *t* test shows the significant difference between mutant and control. *P*-value = 0.0025. Scale bars = 25 µm.

Gene Ontology terms for RNA processing and stress pathways were strongly associated with all differentially expressed genes (Fig. 5e, Supplementary Data 3, 4), while RNA-binding proteins were among the most prominent transcripts deregulated by *Chd7* deletion (Fig. 6a, b). Interestingly, previous reports showed that Chd7 binds to many of their promoters in neuronal progenitors (Fig. S7e, Supplementary Data 9; ref. ^7^) suggesting that they may be direct Chd7 targets. RNA-binding proteins are critical regulators of cellular stress, controlling the assembly and disassembly of stress granules^31–33^. As transient membrane-less compartments, they assemble in the cytoplasm under oxidative stress conditions to allow cells to survive, while their prolonged persistence triggers apoptosis^31–35^. The metabolic demands of sound detection and amplification elicits oxidative stress in neurons and hair cells that causes cell death unless tightly regulated^16,36^. We, therefore, tested the hypothesis that *Chd7* mutant hair cells are hypersensitive to oxidative stress by exploiting a cochlear explant system in which oxidative stress can be induced by treatment with aminoglycosides^37–39^. When P6 control explants from *Atoh1*^*Cre/+*^*;mTmG* mice were exposed to gentamicin for 5 h (100 µM), hair cells were intact along the entire length of the cochlea, as were untreated *Atoh1*^*Cre/+*^*;Chd7*^*f/f*^ homozygous mutant hair cells (*n* = 5/5 each; Fig. 7a, b). In contrast, gentamicin treatment of *Atoh1*^*Cre/+*^*;Chd7*^*f/f*^ mutant explants resulted in a reduction of hair cells by more than 50% across all regions of the cochlea (*n* = 6/6; Fig. 7a, b). These findings show that *Chd7* mutant hair cells are hypersensitive to oxidative stress, causing degeneration in response to stress inducers. Thus, in vivo sound exposure at the onset of hearing may trigger cell death in Chd7-deficient hair cells. Our data suggest that SNHL in CHARGE syndrome may partly be due to misregulation of RNA-binding proteins as key regulators of stress granules thereby altering the response of neurons and hair cells to normal sound.

## Discussion

In humans, *CHD7* haploinsufficiency is the major cause of CHARGE syndrome, a complex developmental disorder associated with growth retardation and defects of the eye, heart, genitals and the ear^1,2^. 50–70% of CHARGE patients present with sensorineural hearing loss, however, the cellular and molecular mechanisms involved are poorly understood. Here we demonstrate that Chd7 plays an essential role in the survival of neurons and hair cells in the murine inner ear. Deleting Chd7 at early developmental stages, when hair cells or neurons are being specified and start to differentiate, does not result in overt developmental phenotypes but rather has longlasting effects on their ability to survive postnatally. What are the underlying molecular mechanisms? Other members of the Chd family are important components in DNA damage response and repair pathways; relocating dynamically to sites of DNA damage, they play critical roles in cell survival^40–43^. The fact that Chd7 function is critical during early embryonic development long before hair cell and neuronal degeneration argues against a direct role for Chd7 in these programmes. It is more likely that Chd7 is required to maintain an epigenomic state that allows a balanced response when exposed to oxidative stress. In support of this idea, we show that loss of Chd7 predisposes embryonic cells to cell death. In the absence of Chd7 function, hair cells are hypersensitive to oxidative stress and key components of these stress response pathways are misregulated. In neuronal progenitor cells, their promoters are occupied by Chd7 suggesting that embryonic loss of Chd7 leads to prolonged epigenomic changes, ultimately preventing the execution of efficient survival programmes necessary to counter oxidative stress. Our findings, therefore, provide a possible explanation and molecular mechanism for SNHL in CHARGE syndrome patients and may pave the way for new therapeutic interventions.

A recent study corroborates our finding that *Atoh1*^*Cre/+*^*;Chd7*^*f/f*^ mutant hair cells develop with seemingly normal morphology at early postnatal stages^44^. We show that from the second postnatal week when hair cells begin to dectect sound and encounter oxidative stress^14,18,19,29,30,35,39^, they rapidly degenerate. While a critical role for Chd7 in promoting neural progenitor expansion has been demonstrated using *Neurog1*^*Cre/+*^*;Chd7*^*f/f*^ mutants, the deletion of Chd7 using *Shh*^*Cre/+*^ in maturing spiral ganglion neurons has no effect on their ongoing development, at least until P1^44^. Whether abnormalities in spiral ganglion neurons arise thereafter is unknown. Our results show that despite normal morphology at P1 in *NeuroD1*^*Cre/+*^*;Chd7*^*f/f*^ mutants, rapid neurodegeneration is observed between P2-P7. This is a crucial and metabolically demanding period for neurons as they establish synaptic connections with hair cells and refine their activity in preparation for the onset of hearing (i.e., P10+)^14,29,30^.

Single-cell transcriptomic data reveal that type I spiral ganglion neurons can be divided into molecularly distinct subtypes^45–47^. Identifying whether specific subtypes undergo neurodegeneration following Chd7 deletion and gaining insights into the disruption of tonotopy may guide improvements in cochlear implants for CHARGE syndrome patients. Interestingly, ~50% of *NeuroD1*^*Cre/+*^*;Chd7*^*f/f*^ mutant spiral ganglion neurons escape degeneration. Although neurons project to the cochlear epithelium and navigate towards hair cells, our transcriptomic data indicate that subtle axonal phenotypes may contribute to hearing loss since a number of genes encoding proteins involved in axon guidance, axon elongation and fasciculation are altered in the mutants.

In summary, Chd7 emerges as a key coordinator of cellular stress proteins. Its embryonic deletion leads to an imbalance in stress pathways that does not affect the normal development of neurons or hair cells. Axons navigate towards hair cells and ribbon synapses are correctly established. However, as cells mature and encounter environmental stress, they begin to degenerate. Our findings suggest that some neurodegenerative diseases arise from neurodevelopmental abnormalities that go undetected, and that SNHL may be an early indicator for these conditions.

## Methods

### Animals

The *Atoh1*^*Cre*^ *(*B6.Cg-Tg(Atoh1-cre)1Bfri*)*^48^*, Chd7*^*flox*^ *(*B6.tm1c(EUCOMM)Wtsi*)*^8^ and *NeuroD1*^*Cre*^ (B6.Cg-Tg(NeuroD1-cre)RZ24Gsat^49^ mice were maintained on a C57BL/6J genetic background. The *mTmG (tm4(ACTB-tdTomato,-EGFP)Luo)*^50^ mice were maintained on a 129S6/SvEv background. The *Atoh1*^*CreERT2*^ (Tg(Atoh1-cre/Esr1*)14Fsh, JAX #007684) used in Fig. S4 was maintained on an FVB/NJ background. The *Chd7*^*floxed*^ mice were crossed with the relevant Cre/reporter lines and backcrossed to C57BL/6J for three generations. All mice were maintained in either C57BL/6J or mixed genetic background. For tamoxifen-induced Cre recombination, a single dose of 20 mg/ml tamoxifen (Sigma, T5648) dissolved in corn oil (Sigma, C8267) was administered to pregnant *Atoh1*^*CreERT2*^;*Chd7*^*flox*^*;mTmG* dams (80 mg/kg of body weight) by intraperitoneal injection. To minimise abortion, 10 mg/ml of progesterone (Sigma: P0130) was simultaneously administered at half tamoxifen dose (40 mg/kg of body weight). One injection gave a consistent recombination efficiency of 95–99%. Upon Cre-mediated recombination, targeted cells expressed membrane GFP. All animal work was performed in accordance with UK Home Office regulations. Experiments were performed on male and female littermates maintained in the same environment to avoid bias. Animals were randomly allocated to experimental groups.

### Immunohistochemistry

Dissected inner ear tissue was fixed in 4% paraformaldehyde (PFA) in phosphate-buffered saline (PBS) and processed for whole-mount immunostaining or frozen sectioning. For whole-mount immunostaining, following permeabilisation with 0.2% Triton X-100/PBS (3 × 10 m) and blocking with 0.2% Triton X-100/5% serum/PBS (1 h), the cochleae were incubated overnight at 4 °C in primary antibodies and then washed in 0.2% Triton X-100 (3 × 10 m). Fluorescent secondary antibodies were applied for 1 h at room temperature. After staining with DAPI, the cochleae were washed extensively prior to mounting onto slides in 50% glycerol/PBS. For immunostaining on cryoprotected sections, following washes in PBS (2 × 10 m), permeabilisation in 0.1% TritonX-100/PBS (1 × 10 m) and blocking in 0.1% TritonX-100/5% serum/PBS (30 m), sections were incubated overnight at 4 °C with primary antibodies. After several washes in 0.1% TritonX-100/PBS, the sections were incubated for 1 h at room temperature with fluorescent secondary antibodies, subsequently washed in PBS, stained with DAPI and mounted onto slides with 50% glycerol/PBS. Primary antibodies used were: rabbit Myo7a (1:1000, Proteus, 25-6790); rabbit NeuN (1:1000, Abcam, ab177487); mouse NF-M (1:200, ThermoFisher Scientific, 13-0700); rabbit Sptbn1 (1:500, Bethyl Laboratories, A300-936A); rabbit Lmx1a (1:100, Abcam, ab139726); rabbit Epha3 (1:100, St John’s Laboratory, STJ110712); mouse Calbindin (1:50, Abcam, ab82812); mouse Parvalbumin (1:100, Sigma, P3088); mouse Satb2 (1:100, Abcam, ab51502); rabbit Chd7 (1:100, ThermoFisher Scientific, PA5-72964); mouse CtBP2 (1:100, BD Biosciences, 612044). Secondary antibodies were: goat anti-rabbit Alexa Fluor 635 (1:500, Invitrogen, A31576); goat anti-mouse Alexa Fluor 488 (1:1000, Invitrogen, A11001). F-actin were stained with Phalloidin 488 (1:1000, Invitrogen, A12379) or 546 (1:500, Invitrogen, A22283).

### In-situ hybridisation

E16.5 inner ears were fixed in 4% PFA overnight at 4 °C followed by cochlea dissection in DEPC-treated PBS. In-situ hybridisation was performed using dig-labelled mouse *Chd7* riboprobe.

### Auditory brainstem response (ABR)

ABR measurements were performed as described in ref. ^51^. An audiometric profile for each mouse at 4 and 8 weeks old was obtained across a range of sound frequencies (3, 6, 12, 18, 24, 30, 36 and 42 kHz). The mice were on a mixed genetic background (C57BL/6J x 129S6/SvEv). Statistical significance was obtained using Kruskal–Wallis non-parametric ANOVA and Bonferroni-corrected significance in GraphPad Prism 9.0.0.121.

### Isolation of hair cells and neurons by FAC-sorting

For RNA-sequencing, samples were collected for three biological replicates on independent occasions. E16.5 cochlear duct from *Atoh1Cre;Chd7flox* mice or P4 spiral ganglia neurons from *NeuroD1Cre;Chd7flox* mice were isolated from inner ears in cold L-15 medium (Thermofisher, 21083027). Tissues were cut into 3–6 pieces depending on stage and collected into low-binding tubes with L-15 on ice. Per experiment, a total of 6 cochleae or ganglia from three siblings were pooled into one tube. Excess L-15 was removed and 100 µl of 20 U/ml Papain (27 mg/ml, Sigma, P3125) and 1 U/ul RNase-free DNAse (Promega, M6101) in L-15 medium was added to each tube. Cells were dissociated at 37 °C in a heated shaker, triturating using a filtered low-binding tip (Alpha Laboratories, LP200NFRS) every 5 m for a total of 40 m for hair cells and 1 h for neurons. The dissociation reaction was stopped by adding 1:1 volume of prewarmed sample buffer (1% fetal bovine serum in L-15). Cells were strained using a 40 µm nylon sterile cell strainer (Falcon, 352340) into a 50 ml low-binding tube (VWR, 5250403) and transferred to a 5 ml FACS tube (Falcon, 352235). DAPI (1 mg/ml) was added (1:1000) immediately prior to FAC-sorting using the BD FACSAria sorters into 1.5 ml low-binding tubes with 100 µl of sample buffer. FAC-sorted cells were centrifuged at 4 °C for 4 m at 8000 relative centrifugal force (Eppendorf centrifuge 54415R), frozen in liquid nitrogen and stored at −80 °C or immediately processed for RNA extraction and first strand cDNA synthesis.

### RNA purification, library preparation and RNA Sequencing

FACS-sorted cells were processed using the NEB Monarch kit (T2010S/L) for polyA+ RNA isolation and NEBNext Single Cell/Low Input RNA Library Prep Kit for Illumina (E6420S/L) and NEBNext Multiplex Oligos for Illumina (Index Primers Set 1, E7335S/L) was used for library preparation (as per kit instructions). RNA and cDNA quality were analysed using Agilent Total RNA 6000 Pico or High Sensitivity DNA Assay on a Bioanalyser (Agilent, 2100). Additional library quality control was performed by the Oxford Genomic Centre at the Wellcome Centre for Human Genetics (funded by the Wellcome Trust, grant 203141/Z/16/Z) and sequenced using Illumina HiSeq 4000 75 bp paired-end reads. Following quality control, paired reads were aligned to mouse MM10 genome assembly. Alignment was performed using HiSAT2 version 2.1.0 with the default parameters in Galaxy version 2.1.0^52,53^. To facilitate quantitative gene expression analysis, aligned reads for each sample were counted using featureCounts version1.6.4^54^.

### Differential gene expression analysis

Differential gene expression analysis was performed using DESeq2 version 2.11.40.6, applying parametric fit^55^. Prior to differential gene expression analysis, a number of filters were applied. We considered the RPKM values of genes that are not normally expressed in E16.5 hair cells (i.e., *Satb2*) or P4 spiral ganglia neurons (i.e., *Atoh1*) and removed all genes with an RPKM value equivalent to or less than *Satb2* or *Atoh1*. This resulted in 6910 transcripts for genes expressed in hair cells and 11,293 genes expressed in neurons. We performed a pairwise comparison between controls and homozygotes and controls and heterozygotes. Considering an adjusted *p*-value (FDR) of ≤0.05 and linear fold change of >2 in either direction, we found a total of 2437 genes in hair cells (1014 upregulated, 1423 downregulated) and 1653 genes in neurons (609 upregulated, 1044 downregulated) that were differentially expressed in *Chd7* homozygous mutants compared to controls (Fig. 3b, Supplementary Data 1–4, see Fig. S7a–d for heterozygotes).

Gene Ontology and Disease Ontology analysis were performed both separately and together on up- or downregulated genes using the R interface (https://cran.r-project.org/web/packages/enrichR/vignettes/enrichR.html) to the Enrichr (https://maayanlab.cloud/Enrichr/) databases (see Supplementary Data 5–8 for specific databases for each analysis). Heatmaps and bubble plots were generated with the R packages *pheatmap* and *GOplot*. Volcano plots were generated in GraphPad Prism 9.0.0.121.

### Quantitative (q) RT-PCR

cDNA from RNA extracted from FAC-sorted hair cells or neurons were subjected to qPCR analysis with the AriaMx Real-Time PCR System (Agilent Technologies) using SYBR green and gene specific primers. Reactions were repeated in triplicates. Relative expression levels were calculated using 2^−ΔΔCT^ method using *Ppp6r3* as an endogenous housekeeping gene. Differences between experimental groups were compared using an unpaired two-tailed Student’s *t*-test and *P*-value ≤0.05 was considered statistically significant.

### Cochlear explants

Cochlear explants from postnatal day 6 *Atoh1*^*Cre/+*^*;mTmG* and *Atoh1*^*Cre/+*^*;Chd7*^*f/f*^*;mTmG* mice were cultured in MatTek dishes coated with CellTak (BD Biosciences). Culture medium comprised of L-15 medium (Thermofisher, 21083027), 5% FBS, 0.2% N2, and 0.001% ciprofloxacin. Explants were maintained at 37 °C under 5% CO_2_ for 1 h prior to gentamicin exposure. Explants were incubated with or without 100 µM gentamicin for 5 h. At 5 h, explants were rinsed in PBS, fixed in 4% PFA (20 m at room temperature) and rinsed again in PBS (3 × 10 m) prior to immunohistochemistry. Experiments were performed on explants of basal and middle turns of the cochlea.

### FM1-43 uptake by hair cells

Cochleae from postnatal day 3 *Atoh1*^*Cre/+*^ (or *Chd7*^*f/f*^) and *Atoh1*^*Cre/+*^*;Chd7*^*f/f*^ mice were dissected in L-15 medium, placed on MatTek dishes and incubated in FM1-43 (Invitrogen) diluted to 3 µM in L-15 for 30 s. Following repeated washes (6×) with L-15, the explants were imaged live under confocal microscopy.

### Microscopy and Imaging

Confocal z stack images were obtained using a TCS SP5 confocal (Leica) microscope, projected using Fiji and further processed using Photoshop (Adobe). Figures were assembled in Photoshop.

### Statistics and reproducibility

Statistical significance for hair cell and neuronal quantification was obtained by performing one-way ANOVA and Dunnett’s multiple comparison test or paired *t*-test. Differences between experimental groups in Fig. 5 were compared using two-tailed unpaired *t*-tests. All statistical tests were conducted using Microsoft Excel and GraphPad Prism 9.0.0.121. Further detail on statistical analyses including sample size and replicates are provided in the relevant method sections and figure legends. Sample size (*n* = 7) is based on power calculations, with an effect size of 1.75, a power of 0.8 and significance level of 0.05.

### Reporting summary

Further information on research design is available in the Nature Research Reporting Summary linked to this article.

## Supplementary information

Supplementary information

Description of Additional Supplementary Files

Supplementary data

Reporting summary

## Data Availability

Sequencing data that support the findings of this study have been deposited in Gene Expression Omnibus with the accession code GSE163798. Source data underlying graphs and charts are provided in Supplementary Data 10.
