# Supplementary material for: The chromatin remodelling factor Chd7 protects auditory neurons and sensory hair cells from stress-induced degeneration
Source: Commun Biol. 2021 Nov 3;4:1260. doi: 10.1038/s42003-021-02788-6 (PMC8566505; doi:10.1038/s42003-021-02788-6)
Supplement: Supplementary file 2 — Description of Additional Supplementary Files [file 42003_2021_2788_MOESM2_ESM.pdf]

Description of Additional Supplementary Files

File name: Supplementary Data
Description:

Supplementary Data 1: FAC-sorted hair cell RNA sample, library data and RPKM values.

Supplementary Data 2: Summary of differential gene expression (hair cells).

Supplementary Data 3: FAC-sorted spiral ganglia neurons RNA sample, library data and RPKM values.

Supplementary Data 4: Summary of differential gene expression (neurons).

Supplementary Data 5: Hair cells: Disease Ontology.

Supplementary Data 6: Neurons: Disease Ontology.

Supplementary Data 7: Hair cells: Gene Ontology.

Supplementary Data 8: Neurons: Gene Ontology.

Supplementary Data 9: Chd7 ChIP-seq (neural progenitors).

Supplementary Data 10:

Source data for graphs and charts:
Figure 2c: Ribbon synapses.
Figure 3n: Hair cell phenotype.
Figure 3o: Neuronal phenotype.
Figure 4: ABR.
Figure 5c: qPCR.
Figure 7b: Oxidative stress-induced hair cell degeneration.
