# Supplementary material for: The chromatin remodelling factor Chd7 protects auditory neurons and sensory hair cells from stress-induced degeneration
Source: Commun Biol. 2021 Nov 3;4:1260. doi: 10.1038/s42003-021-02788-6 (PMC8566505; doi:10.1038/s42003-021-02788-6)
Supplement: Supplementary file 4 — Reporting summary [file 42003_2021_2788_MOESM4_ESM.pdf]

nature portfolio

Corresponding author(s): Andrea Streit & Mohi Ahmed

Last updated by author(s): Oct 4, 2021

Reporting Summary

Nature Portfolio wishes to improve the reproducibility of the work that we publish. This form provides structure for consistency and transparency in reporting. For further information on Nature Portfolio policies, see our Editorial Policies and the Editorial Policy Checklist.

Statistics

For all statistical analyses, confirm that the following items are present in the figure legend, table legend, main text, or Methods section.

n/a	Confirmed	
☐	☒	The exact sample size (*n*) for each experimental group/condition, given as a discrete number and unit of measurement
☐	☒	A statement on whether measurements were taken from distinct samples or whether the same sample was measured repeatedly
☐	☒	The statistical test(s) used AND whether they are one- or two-sided<br>*Only common tests should be described solely by name; describe more complex techniques in the Methods section.*
☒	☐	A description of all covariates tested
☒	☐	A description of any assumptions or corrections, such as tests of normality and adjustment for multiple comparisons
☐	☒	A full description of the statistical parameters including central tendency (e.g. means) or other basic estimates (e.g. regression coefficient) AND variation (e.g. standard deviation) or associated estimates of uncertainty (e.g. confidence intervals)
☒	☐	For null hypothesis testing, the test statistic (e.g. *F*, *t*, *r*) with confidence intervals, effect sizes, degrees of freedom and *P* value noted<br>*Give P values as exact values whenever suitable.*
☒	☐	For Bayesian analysis, information on the choice of priors and Markov chain Monte Carlo settings
☒	☐	For hierarchical and complex designs, identification of the appropriate level for tests and full reporting of outcomes
☒	☐	Estimates of effect sizes (e.g. Cohen's *d*, Pearson's *r*), indicating how they were calculated

Our web collection on statistics for biologists contains articles on many of the points above.

Software and code

Policy information about availability of computer code

Data collection	RNA sequenced using Illumina HiSeq 4000 75bp paired-end reads.
Data analysis	Paired sequencing reads were aligned to mouse MM10 genome assembly using HiSAT2 version 2.1.0 with the default parameters in Galaxy version 2.1.0 and counted using featureCounts version1.6.4. Differential gene expression analysis was performed using DESeq2 version 2.11.40.6, applying parametric fit. R packages pheatmap, GOplot and Enrichr databases were used to generate heatmaps, bubble plots and ontologies using RStudio version 1.3.1073. Volcano plots were generated in GraphPad Prism 9.0.0.121. All statistical tests were conducted using Microsoft Excel version 2002 and/or GraphPad Prism version 9.0.0.121. Confocal z stack images were obtained using a TCS SP5 confocal (Leica) microscope, projected using Fiji and further processed using Photoshop (Adobe).

For manuscripts utilizing custom algorithms or software that are central to the research but not yet described in published literature, software must be made available to editors and reviewers. We strongly encourage code deposition in a community repository (e.g. GitHub). See the Nature Portfolio guidelines for submitting code & software for further information.

Data

Policy information about availability of data

All manuscripts must include a data availability statement. This statement should provide the following information, where applicable:

- Accession codes, unique identifiers, or web links for publicly available datasets
- A description of any restrictions on data availability
- For clinical datasets or third party data, please ensure that the statement adheres to our policy

Sequencing data that support the findings of this study have been deposited in Gene Expression Omnibus with the accession code GSE163798. Source data underlying graphs and charts are provided in Supplementary Data 10.

Field-specific reporting

Please select the one below that is the best fit for your research. If you are not sure, read the appropriate sections before making your selection.

☒ Life sciences ☐ Behavioural & social sciences ☐ Ecological, evolutionary & environmental sciences

For a reference copy of the document with all sections, see nature.com/documents/nr-reporting-summary-flat.pdf

Life sciences study design

All studies must disclose on these points even when the disclosure is negative.

Sample size	Power calculations were used to estimate the sample size (i.e., n=7) and significance with a two-tailed t-test. For ethical applications, we specified a 'large effect size of 1.7 (calculated based on preliminary data where n=7, power 0.85, significance 0.05 = effect size of 1.75, d of 3.27 and t of 2.18), a power of 0.8, a significance level of 0.05 in the G* Power statistical software for calculations (Noncentrality parameter d = 3.18; Critical t = 2.12; Df = 12; Sample size = 7; Actual power = 0.83).
Data exclusions	Samples for which we could not verify the genotype were excluded from our analysis.
Replication	We could successfully replicate our data - experiments were repeated three times or more.
Randomization	Mice were randomly assigned to different test groups and maintained in the same environment to avoid bias.
Blinding	Investigators were blinded to ABR tests and some phenotypic analysis but not all, including explants where it was necessary to group samples according to genotype prior to drug treatment.

Reporting for specific materials, systems and methods

We require information from authors about some types of materials, experimental systems and methods used in many studies. Here, indicate whether each material, system or method listed is relevant to your study. If you are not sure if a list item applies to your research, read the appropriate section before selecting a response.

Materials & experimental systems

n/a	Involved in the study	
☐	☒	Antibodies
☒	☐	Eukaryotic cell lines
☒	☐	Palaeontology and archaeology
☐	☒	Animals and other organisms
☒	☐	Human research participants
☒	☐	Clinical data
☒	☐	Dual use research of concern

Methods

n/a	Involved in the study	
☒	☐	ChIP-seq
☐	☒	Flow cytometry
☒	☐	MRI-based neuroimaging

Antibodies

Antibodies used	rabbit Myo7a (1:1000, Proteus, 25-6790)<br>rabbit NeuN (1:1000, Abcam, ab177487)<br>mouse NF-M (1:200, ThermoFisher Scientific, 13-0700)<br>rabbit Sptbn1 (1:500, Bethyl Laboratories, A300-936A)<br>rabbit Lmx1a (1:100, Abcam, ab139726)<br>rabbit Epha3 (1:100, St John's Laboratory, STJ110712)<br>mouse Calbindin (1:50, Abcam, ab82812)<br>mouse Parvalbumin (1:100, Sigma, P3088)<br>mouse Satb2 (1:100, Abcam, ab51502)<br>rabbit Chd7 (1:100, ThermoFisher Scientific, PA5-72964)<br>mouse CtBP2 (1:100, BD Biosciences, 612044)<br>goat anti-rabbit Alexa Fluor 635 (1:500, Invitrogen, A31576)<br>goat anti-mouse Alexa Fluor 488 (1:1000, Invitrogen, A11001)
Validation	rabbit Myo7a: Huang et al. Reciprocal Negative Regulation Between Lmx1a and Lmo4 Is Required for Inner Ear Formation. J Neurosci. 38, 5429-5440 (2018); Ahmed, M., Ura, K., & Streit, A. Auditory hair cell defects as potential cause for sensorineural deafness in Wolf-Hirschhorn syndrome. Dis. Model. Mech. 8, 1027-1035 (2015).<br>rabbit NeuN: Abcam – Immunocytochemistry/immunofluorescence analysis of Mouse primary neuron cells labelling NeuN with ab177487 at 1:100.<br>mouse NF-M: Ahmed, M., Ura, K., & Streit, A. Auditory hair cell defects as potential cause for sensorineural deafness in Wolf-Hirschhorn syndrome. Dis. Model. Mech. 8, 1027-1035 (2015).

rabbit Sptbn1: Liu, Y et al. Critical role of spectrin in hearing development and deafness. Sci. Adv. 5, eaav7803 (2019).
rabbit Lmx1a: Huang, Y et al. Reciprocal Negative Regulation Between Lmx1a and Lmo4 Is Required for Inner Ear Formation. J Neurosci. 38, 5429-5440 (2018).
rabbit Epha3: St John's Laboratory – Immunohistochemistry of paraffin-embedded rat brain using EPHA3 antibody (STJ110712) at dilution of 1:100.
goat anti-rabbit Alexa Fluor 635: Ahmed, M & Streit, A. Lsd1 interacts with cMyb to demethylate repressive histone marks and maintain inner ear progenitor identity. Development. 145, dev160325 (2018).
goat anti-mouse Alexa Fluor 488: Ahmed, M., Ura, K., & Streit, A. Auditory hair cell defects as potential cause for sensorineural deafness in Wolf-Hirschhorn syndrome. Dis. Model. Mech. 8, 1027-1035 (2015).

Animals and other organisms

Policy information about studies involving animals; ARRIVE guidelines recommended for reporting animal research

Laboratory animals: Both male and female mice from predominantly C57BL/6J and mixed C57BL/6J;129S6/SvEv or C57BL/6J;129S6/SvEv;FVB/NJ strains were used in the study. The primary phenotype was consistent in all strains.

Wild animals: The study did not involve wild animals.

Field-collected samples: The study did not involve samples collected from the field.

Ethics oversight: All animal work was performed in accordance with King's College London animal welfare and UK Home Office regulations.

Note that full information on the approval of the study protocol must also be provided in the manuscript.

Flow Cytometry

Plots

Confirm that:

☒ The axis labels state the marker and fluorochrome used (e.g. CD4-FITC).

☒ The axis scales are clearly visible. Include numbers along axes only for bottom left plot of group (a 'group' is an analysis of identical markers).

☒ All plots are contour plots with outliers or pseudocolor plots.

☒ A numerical value for number of cells or percentage (with statistics) is provided.

Methodology

Sample preparation: Samples were collected for three biological replicates on independent occasions. Cochlear duct or spiral ganglia neurons were isolated from inner ears in cold L-15 medium (Thermofisher, 21083027). Tissues were cut into 3-6 pieces depending on stage and collected into low-binding tubes with L-15 on ice. Per experiment, a total of 6 cochleae or ganglia from three siblings were pooled into one tube. Excess L-15 was removed and 100µl of 20U/ml Papain (27mg/ml, Sigma, P3125) and 1U/ul RNase-free DNAse (Promega, M6101) in L-15 medium was added to each tube. Cells were dissociated at 37C in a heated shaker, triturating using a filtered low-binding tip (Alpha Laboratories, LP200NFRS) every 5 minutes for a total of 40 minutes for hair cells and 1 hour for neurons. The dissociation reaction was stopped by adding 1:1 volume of prewarmed sample buffer (1% fetal bovine serum in L-15). Cells were strained using a 40µm nylon sterile cell strainer (Falcon, 352340) into a 50ml low-binding tube (VWR, 5250403) and transferred to a 5ml FACS tube (Falcon, 352235). DAPI (1mg/ml) was added (1:1000) immediately prior to FAC-sorting using the BD FACSAria sorters into 1.5ml low-binding tubes with 100µl of sample buffer. FAC-sorted cells were centrifuged at 4C for 4 minutes at 8000 relative centrifugal force (Eppendorf centrifuge 54415R), frozen in liquid nitrogen and stored at -80C or immediately processed for RNA extraction and first strand cDNA synthesis.

Instrument: BD FACSAria sorter

Software: BD FACSDiva 8.0.1

Cell population abundance: Cell-type purity was determined by fluorescence-tagged lineage tracing (tomato and GFP fluorescence) - only GFP and double positive cells were sorted for analysis while red-only cells were discarded. An average of 800 purified hair cells or 7000 spiral ganglia neurons were obtained from each inner ear.

Gating strategy: Unstained and DAPI stained samples were used to discriminate the boundary between live/dead cells. Cre-recombined cells were identified as GFP only or double positive (GFP+, tomato+) and gated separately from non-target (tomato+ only) cells.

☒ Tick this box to confirm that a figure exemplifying the gating strategy is provided in the Supplementary Information.

March 2021
